# Advanced TiO_2_-Based Photocatalytic Systems for Water Splitting: Comprehensive Review from Fundamentals to Manufacturing

**DOI:** 10.3390/molecules30051127

**Published:** 2025-02-28

**Authors:** Tarek Ahasan, E. M. N. Thiloka Edirisooriya, Punhasa S. Senanayake, Pei Xu, Huiyao Wang

**Affiliations:** Department of Civil Engineering, New Mexico State University, Las Cruces, NM 88003, USA; tarek@nmsu.edu (T.A.); thiloka@nmsu.edu (E.M.N.T.E.); punhasa@nmsu.edu (P.S.S.); pxu@nmsu.edu (P.X.)

**Keywords:** photocatalytic water splitting, hydrogen evolution, heterojunction engineering, water chemistry, scale-up engineering, reactor design, artificial intelligence in catalysis, sustainable energy

## Abstract

The global imperative for clean energy solutions has positioned photocatalytic water splitting as a promising pathway for sustainable hydrogen production. This review comprehensively analyzes recent advances in TiO_2_-based photocatalytic systems, focusing on materials engineering, water source effects, and scale-up strategies. We recognize the advancements in nanoscale architectural design, the engineered heterojunction of catalysts, and cocatalyst integration, which have significantly enhanced photocatalytic efficiency. Particular emphasis is placed on the crucial role of water chemistry in photocatalytic system performance, analyzing how different water sources—from wastewater to seawater—impact hydrogen evolution rates and system stability. Additionally, the review addresses key challenges in scaling up these systems, including the optimization of reactor design, light distribution, and mass transfer. Recent developments in artificial intelligence-driven materials discovery and process optimization are discussed, along with emerging opportunities in bio-hybrid systems and CO_2_ reduction coupling. Through critical analysis, we identify the fundamental challenges and propose strategic research directions for advancing TiO_2_-based photocatalytic technology toward practical implementation. This work will provide a comprehensive framework for exploring advanced TiO_2_-based composite materials and developing efficient and scalable photocatalytic systems for multifunctional simultaneous hydrogen production.

## 1. Introduction

**Figure 1:**
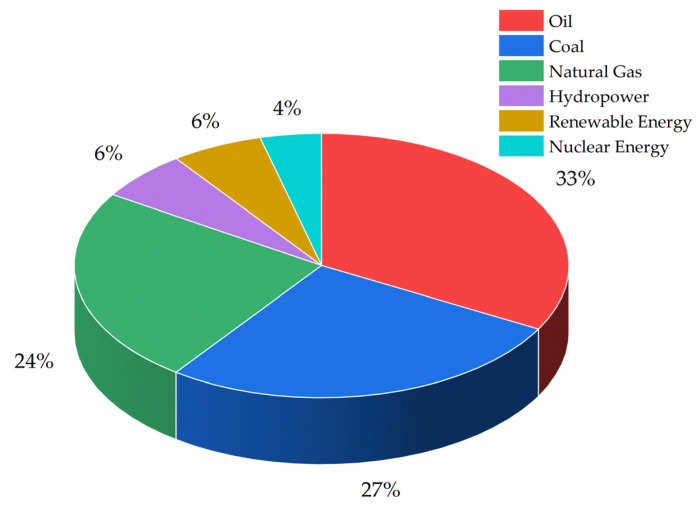
Global energy resource distribution by percentage [25].

Energy is becoming an increasingly critical factor in global socio-economic development and environmental sustainability [1,2,3]. The global energy infrastructure continues to be overwhelmingly reliant on fossil fuels, with oil being the predominant source, followed closely by coal, while natural gas constitutes approximately one-fourth of power generation (Figure 1) [4,5]. This predominant reliance on non-renewable energy sources has precipitated substantial environmental deterioration through carbon dioxide emissions (CO_2_), exacerbating both concerns about climate change and the challenges inherited with global energy security [6,7,8]. These global challenges represent two of humanity’s most pressing concerns, particularly in light of the IPCC’s recommendation to achieve substantial reductions in CO_2_ emissions by 2050 and limit the global average temperature increase to below 1.5 °C [9,10,11,12]. To meet these ambitious zero-emission targets while ensuring energy security, a transition toward clean, sustainable energy alternatives is imperative [13,14]. Among the various renewable energy sources, hydrogen has emerged as a promising energy carrier due to its zero greenhouse gas emissions, superior energy capacity, and ecological sustainability [15,16,17,18]. However, the current hydrogen production methods, dominated by steam methane reforming, contribute significantly to greenhouse gas emissions, accounting for approximately 530 million tons of CO_2_ annually [19,20,21]. While hydrogen can be produced through multiple pathways, photocatalytic water splitting using natural light has recently gained attention as a sustainable approach that harnesses abundant renewable resources such as water and solar energy [22,23,24].

Photocatalytic water splitting represents a transformative approach to hydrogen production, offering the possibility of harnessing abundant solar energy to generate clean hydrogen fuel [23,26,27]. Among the various photocatalytic materials, titanium dioxide (TiO_2_) has maintained its position as a cornerstone material since Fujishima and Honda’s seminal work in 1972 [28,29]. The enduring interest in TiO_2_-based systems stems from their unique combination of chemical stability, cost-effectiveness, low toxicity, and favorable band edge positions for water splitting reactions [30,31,32,33].

Recent years have witnessed remarkable advances in TiO_2_-based photocatalytic and photo-reforming systems [34,35,36,37,38,39,40]. The emergence of precise nanoscale engineering techniques has enabled unprecedented control over material properties, leading to significant improvements in hydrogen evolution rates [41,42,43]. Notable achievements include the development of black TiO_2_ with engineered oxygen vacancies, showing an exceptional hydrogen evolution performance under visible light irradiation, and the creation of Z-scheme TiO_2_/g-C_3_N_4_ heterojunction systems, demonstrating promising photocatalytic activity with a superior quantum efficiency under solar illumination [44,45,46,47,48]. Additionally, noble-metal-decorated TiO_2_ nanomaterials with an optimized morphology have exhibited a remarkable stability over extended periods while maintaining outstanding hydrogen production rates [49,50,51]. 

Despite these significant advances, several fundamental challenges continue to impede the widespread implementation of TiO_2_-based photocatalytic systems for practical hydrogen production. The primary limitations include the wide bandgap of TiO_2_ (3.2 eV), which restricts light absorption to the UV region, representing only about 4% of the solar spectrum [34,52,53,54]. Therefore, pristine TiO_2_ exhibits minimal hydrogen production under natural light conditions due to the low intensity of UV light in the solar spectrum. While various bandgap engineering strategies have been explored, both achieving efficient visible light absorption and maintaining appropriate band positions for water splitting remains a significant challenge, as visible light, which constitutes a larger portion of the solar spectrum than UV, offers more practical and sustainable energy for photocatalytic processes [55,56]. Another critical barrier is the rapid recombination of photogenerated electron–hole pairs, which substantially reduces quantum efficiency [57,58,59]. Although heterojunction engineering and cocatalyst incorporation have shown promise in charge carrier separation, the fundamental understanding of interfacial charge transfer dynamics and the precise role of cocatalysts in multi-component systems requires further investigation [60,61,62]. The stability of these complex architectures under prolonged photocatalytic conditions, particularly in terms of maintaining interfacial contacts and preventing photocorrosion, presents additional challenges [63,64,65]. Water chemistry also emerges as a critical yet often overlooked factor in photocatalytic hydrogen production efficiency. The pH, ionic strength, and dissolved species in the reaction medium significantly influence the surface chemistry of TiO_2_ and the kinetics of water splitting reactions [66,67,68,69,70]. Moreover, hardness and impurities in the water can lead to surface poisoning or competitive reactions that diminish hydrogen evolution rates [71,72]. These challenges become particularly pronounced when considering alternative water sources such as municipal wastewater, seawater, and produced water from industrial processes [73,74,75,76]. While these non-traditional water sources present an attractive opportunity for sustainable hydrogen production, their complex chemical matrices (such as total dissolved solids concentrations ranging from ~500 mg/L of municipal wastewater to 260,000 mg/L in oil-field-produced water) introduce additional complications, including enhanced catalyst deactivation and selective ion interference [77,78,79]. Understanding the intricate relationships between water source characteristics and photocatalytic performance is crucial for advancing the field toward practical applications.

This comprehensive review provides a holistic and multidisciplinary perspective on TiO_2_-based photocatalytic hydrogen production, bridging fundamental materials advancements, water chemistry effects, and large-scale engineering challenges. While previous reviews have largely focused on either photocatalyst development or reaction mechanisms, this work distinguishes itself by systematically examining the often-overlooked role of water chemistry and real-world water sources—including municipal wastewater, seawater, and industrial effluents—highlighting their direct influence on photocatalytic performance, catalyst stability, and process optimization. Additionally, this review presents an in-depth discussion on scale-up strategies, including reactor design optimization, light distribution, mass transfer phenomena, and techno-economic considerations, which are crucial for transitioning from laboratory research to industrial application. Beyond traditional material science perspectives, this review also explores AI-driven material discovery for catalyst optimization, bio-hybrid photocatalytic systems for enhanced efficiency, and the integration of CO_2_ reduction pathways to improve sustainability. By integrating these diverse yet interconnected aspects, this work provides a strategic framework for advancing scalable, stable, and economically viable photocatalytic water splitting technologies for sustainable hydrogen production.

## 2. Mechanism of Photocatalytic Water Splitting

Photocatalytic water splitting represents a sophisticated approach for converting light energy into chemical energy through water decomposition into stoichiometric hydrogen and oxygen using photocatalysts [80,81]. This process fundamentally relies on semiconductor photocatalysts, where incident photons generate electron–hole pairs by exciting the electron when the light energy equals or exceeds the photocatalyst’s bandgap energy [82,83]. The mechanism follows thermodynamic constraints, requiring a Gibbs free energy change of 237 kJ/mol (ΔG = +237 kJ/mol) and an energy barrier of 1.23 eV for the complete water splitting redox process [84,85].

**Figure 2:**
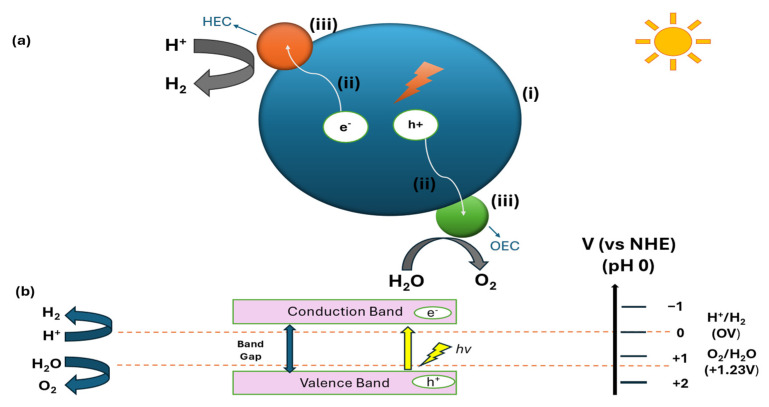
(**a**) Schematic representing the key steps of photocatalytic water splitting: light absorption, charge carrier separation, and surface redox reactions. (**b**) Energy band diagram illustrating the thermodynamic requirements for water splitting using semiconductor photocatalysts [85].

The following two distinct mechanisms have emerged for photocatalytic water splitting: the one-step and two-step (Z-scheme) photo-excitation processes [86]. In the one-step mechanism (Figure 2), photoexcitation generates electron–hole pairs through the promotion of electrons to the conduction band (CB) while creating positive holes in the valence band (VB) [87]. The efficacy of photocatalytic water splitting hinges on precise energetic alignment—the semiconductor’s CB minimum must maintain a more negative potential than the H^+^/H_2_ reduction potential (0 V vs. Normal Hydrogen Electrode (NHE) at pH = 0), while its VB maximum must exceed the O_2_/H_2_O oxidation potential (+1.23 V vs. NHE at pH = 0) [88]. The efficiency of one-step photocatalytic water splitting is governed by the following three fundamental processes: photon absorption, charge carrier dynamics (separation and transport), and the surface-mediated catalytic reactions of adsorbed species (Figure 2) [89]. In the one-step mechanism, a single photocatalyst directly facilitates water splitting through the following sequential reactions [86]:

Photocatalyst → (e^−^ + h^+^) (1)

Catalyst (e^−^ + h^+^) → Catalyst (2)

hν → 2e^−^ + 2h^+^ (3)

H_2_O (l) + 2h^+^ → ½ O_2_ + 2H^+^ (4)

2H^+^ + 2e^−^ → H_2_(g) (5)

**Figure 3:**
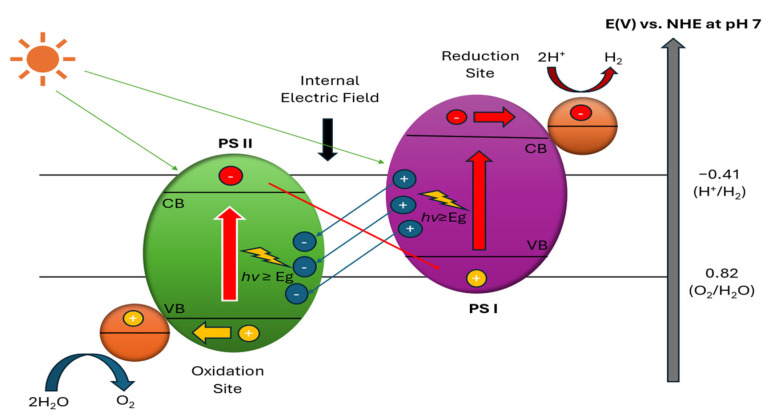
Diagram illustrating the energy band in a two-step photocatalytic water splitting system [101].

The Z-scheme approach (Figure 3) employs dual photocatalysts for hydrogen and oxygen production, linked by electron-shuttling redox mediators [90]. A material qualifies as a hydrogen evolution photocatalyst in Z-scheme systems when its CB minimum lies above the proton reduction potential while its VB maximum exceeds the mediator’s redox potential [91]. A material can function as an oxygen evolution photocatalyst when its VB maximum surpasses water’s oxidation potential, with its CB minimum positioned above the mediator’s redox potential [92,93,94]. The more flexible thermodynamic criteria for Z-scheme systems enable redox reactions between the mediator species and significantly expand the selection of suitable photocatalysts for overall water splitting [95,96,97,98,99,100].

While significant advances have been made in understanding photocatalytic water splitting mechanisms, several fundamental challenges remain unresolved. The current challenges include rapid charge carrier recombination in one-step systems [102,103] and interfacial charge transfer resistance in Z-scheme approaches [104,105,106]. Recent attempts to enhance charge separation through cocatalyst integration have shown limited success, with noble metal cocatalysts improving efficiency by only 2–3 fold while significantly increasing system costs [107,108]. Surface modification strategies, particularly the creation of oxygen vacancies, have demonstrated enhanced visible light absorption but often face stability challenges, as these defects tend to be filled by water/oxygen from the air or are blocked by reaction intermediates, leading to gradual performance decline unless specific stabilization strategies are employed [109,110]. These challenges are particularly evident in TiO_2_-based systems, where the wide bandgap of 3.2 eV limits visible light absorption, while rapid electron–hole recombination and surface stability issues continue to constrain the hydrogen evolution rates.

## 3. Electrochemical Characterization and Hydrogen Production Rate Calculation

### 3.1. Electrochemical Property Analysis

Electrochemical characterization techniques provide crucial insights into the fundamental properties and performance of TiO_2_-based photocatalysts for water splitting applications. These methods assess charge carrier dynamics, catalytic activity, and reaction kinetics, offering quantitative measures of photocatalytic efficiency [111]. Importantly, these electrochemical metrics directly correlate with hydrogen production rates, establishing structure–property–performance relationships essential for catalyst design. These electrochemical characterization methods encompass several complementary techniques that provide a quantitative assessment of photocatalytic performance, as detailed below.

#### 3.1.1. Linear Sweep Voltammetry (LSV)

LSV represents a fundamental electrochemical technique for evaluating the catalytic activity of TiO_2_-based photocatalysts toward the hydrogen evolution reaction (HER) and oxygen evolution reaction (OER) [112]. In a typical LSV measurement, the working electrode’s potential is swept linearly with time while monitoring the current response. This method helps to determine onset potential, which signifies the initiation of the HER or OER. Lower (more negative) onset potentials for HER and higher (more positive) onset potentials for OER reflect a superior catalytic activity [113,114]. Additionally, current density, expressed as mA/cm^2^, is an important parameter that reflects catalytic performance, as higher current densities at a given potential suggest improved reaction kinetics [115]. 

For TiO_2_-based photocatalysts, LSV measurements are conducted both in the dark and under illumination, with the difference (photocurrent) directly correlating with the material’s photoelectrocatalytic activity. The photocurrent onset potential and its magnitude serve as critical parameters for evaluating the photocatalyst efficiency [116,117]. 

#### 3.1.2. Tafel Analysis

Tafel analysis provides mechanistic insights into electrochemical reactions by examining the relationship between overpotential (η) and the logarithm of current density (log|j|), following the Tafel equation [118].

η = a + b log|j| (6)

where b represents the Tafel slope, typically expressed in mV/decade, and a is the intercept related to the exchange current density. 

Different Tafel slope values correspond to distinct electrochemical mechanisms. In TiO_2_-based HER systems, slopes of approximately 120, 40, and 30 mV/decade indicate the Volmer, Heyrovsky, and Tafel mechanisms, respectively [119,120,121]. A lower Tafel slope reflects improved catalytic kinetics, as it suggests a more efficient charge transfer process during hydrogen evolution [119,120,121].

Certain modifications to TiO_2_ photocatalysts, such as integration with noble metal cocatalysts or the introduction of tensile strain, have been shown to reduce Tafel slopes, highlighting improved charge transfer kinetics and enhanced hydrogen evolution rates. Noble metal cocatalysts primarily enhance charge separation and reduce recombination, whereas structural modifications such as tensile strain or oxygen vacancies directly influence charge transfer efficiency and reaction kinetics [122,123]. 

#### 3.1.3. Electrochemical Impedance Spectroscopy (EIS)

EIS provides detailed information about the interfacial charge transfer processes and carrier dynamics [124,125]. EIS measurements are typically represented as Nyquist plots, where the semicircle diameter corresponds to the charge transfer resistance, which is primarily associated with the electrode/electrolyte interface in the medium frequency range. Smaller semicircle diameters indicate a lower charge transfer resistance, reflecting improved charge transfer kinetics [126,127]. For TiO_2_-based photocatalysts, EIS analysis under illumination reveals significant decreases in charge transfer resistance compared to dark conditions, quantifying the photoinduced enhancement of charge carrier transport [111]. Additionally, the equivalent circuit modeling of EIS data provides valuable insights into the following: (1) Double-layer capacitance (Cdl): This parameter reflects the ability of the electrode surface to store charge at the electrode/electrolyte interface, directly correlating with the electrochemically active surface area. A higher Cdl indicates a larger accessible surface area for electrochemical reactions [128]. (2) Charge carrier lifetime (τ): Estimated from the characteristic frequency (fmax) at the maximum phase angle, τ represents the time scale over which charge carriers remain mobile before recombination or trapping. It is calculated using τ = 1/(2πfmax), making it a critical metric for evaluating the efficiency of charge transport in photoelectrochemical and electrocatalytic systems [126]. (3) Series resistance: This represents the ohmic resistance in the system, primarily arising from the electrolyte, electrical contacts, and internal resistances within the electrode. It is determined from the high-frequency intercept of the Nyquist plot and directly impacts the overall system efficiency by influencing potential losses during electrochemical processes [126]. These parameters collectively characterize the efficiency of charge transfer processes in TiO_2_-based photocatalytic systems.

#### 3.1.4. Mott–Schottky Analysis

Mott–Schottky analysis provides essential information about the semiconductor properties of TiO_2_-based photocatalysts, including the determination of the flat-band potential and carrier density [129]. For TiO_2_-based photocatalysts, Mott–Schottky plots typically display positive slopes, confirming n-type semiconductor behavior. This characteristic arises from the formation of a depletion layer at the semiconductor/electrolyte interface, as consistently observed in TiO_2_ thin films, nanotube arrays, and other TiO_2_-based materials [129,130,131]. The flat-band potential is determined from the x-intercept of the Mott–Schottky plot and indicates the potential at which band bending is neutralized. This parameter plays a key role in aligning TiO_2_’s band edges with HER and OER redox potentials, directly affecting its photocatalytic efficiency [132,133,134]. Carrier concentration can be estimated from the slope of the Mott–Schottky plot, where a steeper slope corresponds to a higher charge carrier density. A greater carrier concentration facilitates charge transport, minimizes recombination losses, and enhances overall hydrogen evolution rates [135,136]. 

Modifying TiO_2_ through doping, heterojunction formation, or creating oxygen vacancies significantly enhances its properties as a photocatalyst. These modifications increase the charge carrier concentration, improving electron movement and boosting the hydrogen production efficiency. Studies show that such enhancements can increase the carrier concentration by up to 1–2 orders of magnitude, resulting in a substantial improvement in hydrogen evolution rates compared to unmodified TiO_2_ [137,138]. 

### 3.2. Quantification of Hydrogen Production Rates

The practical evaluation of TiO_2_-based photocatalysts requires the quantification of hydrogen production rates under standardized conditions. Several analytical techniques enable precise hydrogen quantification.

#### 3.2.1. Gas Chromatography

Gas Chromatography provides high-sensitivity hydrogen quantification with detection limits as low as 0.1 μmol, using thermal conductivity detectors for general gas detection and flame ionization detectors (FIDs) with methanizers for enhanced hydrocarbon analysis. Calibration with standard hydrogen concentrations enables precise quantification based on peak areas, ensuring accurate impurity analysis and compliance with hydrogen purity standards [139,140].

#### 3.2.2. Mass Spectrometry 

Mass Spectrometry enables the real-time analysis of evolved hydrogen with a high sensitivity and selectivity through mass-to-charge ratio discrimination. Certain Mass Spectrometry techniques, such as Selected Ion Flow Tube Mass Spectrometry (SIFT-MS) and Time-of-Flight Mass Spectrometry (TOF-MS), enable the real-time analysis of evolved hydrogen with a high sensitivity (detection limits as low as 0.001–0.8 μmol·mol^−1^, depending on the method) and selectivity through mass-to-charge ratio discrimination (GC-MS and EI-MS) or ion-molecule reaction processes (SIFT-MS) [141].

#### 3.2.3. Volumetric Methods

Volumetric methods provide a direct means of measuring gas quantities using liquid displacement techniques, such as Mariotte flasks or precision gas burettes. These methods allow for accurate volume measurement, which is then converted into molar quantities using the ideal gas law, as follows:

(7) $${\mathrm{nH}}_{2}=\frac{PV}{RT}$$

where nH_2_ represents the moles of hydrogen, P is the pressure, V is the volume, R is the universal gas constant, and T is the absolute temperature. This approach enables the precise quantification of hydrogen evolution in laboratory and industrial applications [142]. 

While these quantification methods provide crucial performance metrics for evaluating hydrogen production efficiency, the practical advancement of TiO_2_-based photocatalysts ultimately depends on innovative engineering approaches that address their fundamental limitations and enhance their photocatalytic properties.

## 4. Advanced Engineering of TiO_2_ for Photocatalytic Water Splitting

Recent advances in materials science and nanotechnology have enabled unprecedented control over the structural and electronic properties of TiO_2_-based photocatalysts, leading to significant improvements in water splitting efficiency. These developments encompass multiple engineering strategies, ranging from precise architectural design to sophisticated surface modifications and interface engineering. The following sections will detail these key advances, beginning with fundamental architectural considerations and crystal-phase engineering, followed by surface chemistry modifications, heterojunction development, and cocatalyst integration strategies. Each of these approaches contributes uniquely to addressing the inherent limitations of TiO_2_-based systems, particularly in terms of light absorption, charge separation, and catalytic activity.

### 4.1. Architectural Design and Crystal-Phase Engineering

The evolution of nanoscale material engineering has fundamentally transformed TiO_2_-based photocatalyst design, introducing sophisticated approaches that have revolutionized photocatalytic water splitting efficiency [143,144,145,146,147,148,149,150]. This transformation encompasses multiple critical advances in materials design and engineering, ranging from novel nanostructure architectures to advanced interface engineering strategies, each uniquely contributing to an enhanced photocatalytic performance [151,152,153]. Hierarchical nanostructures have emerged as a groundbreaking development, integrating multiple morphological elements such as nanotubes, nanosheets, and nanoparticles into cohesive architectures [154,155,156]. These sophisticated structures demonstrate remarkable improvements in light harvesting efficiency through enhanced light scattering and trapping mechanisms [157,158]. When combined with carefully engineered macro/mesoporous structures, these systems facilitate superior mass transport while maximizing reactive surface area, leading to significant improvements in photocatalytic activity [159,160,161]. The integration of ordered arrays and self-assembled structures has particularly advanced directional charge transport, addressing one of the fundamental limitations in traditional photocatalyst designs [162,163,164]. Crystal-phase engineering has emerged as a critical strategy in photocatalyst development, with mixed-phase TiO_2_ (anatase/rutile) junctions exhibiting exceptional charge separation properties. The controlled synthesis of these phase junctions has enabled the precise manipulation of electron–hole pair dynamics, significantly reducing recombination rates [165,166,167,168,169,170]. Table 1 summarizes these key advancements in TiO_2_-based photocatalysts, highlighting their features and specific impacts on hydrogen production efficiency via water splitting.

While these architectural advances show promise, each approach presents specific challenges and limitations. Hierarchical nanostructures, despite their enhanced light-harvesting capabilities, often suffer from complex synthesis procedures that can introduce structural defects at interfaces, potentially creating electron–hole recombination centers [176,177]. The precise control required for the optimal performance in these structures presents significant challenges in large-scale manufacturing [176,178]. Macro/mesoporous structures, while offering superior mass transport, can experience pore blocking during extended operation, particularly in real-world conditions where water impurities are present [179]. This limitation necessitates careful consideration of pore size distribution and surface chemistry to maintain long-term stability [180]. Ordered arrays and self-assembled structures face challenges in maintaining their structural integrity during long-term operation, with thermal cycling and mechanical stress potentially disrupting the careful arrangement of components [181]. Moreover, the interface quality between different components in these structures significantly impacts their performance, with poor interfaces acting as recombination sites [182,183]. 

### 4.2. Surface Chemistry and Defect Engineering

Notably, the development of black TiO_2_ through controlled oxygen vacancy engineering has revolutionized visible light absorption capabilities. These modified materials demonstrate photocatalytic activity well beyond traditional UV-restricted domains, with some systems showing remarkable quantum efficiencies under visible light irradiation [184,185]. Surface modification and defect engineering have advanced significantly, with atomic-level control over surface chemistry becoming increasingly precise [186,187]. The strategic introduction of oxygen vacancies and Ti^3+^ states, coupled with non-metal doping, has enabled targeted band structure modifications [188,189,190]. These modifications have substantially improved visible light absorption and charge separation efficiency, with some systems demonstrating a marked increase in photocatalytic hydrogen evolution rates compared to unmodified TiO_2_ [191,192]. Advanced characterization techniques, including in situ X-ray absorption spectroscopy and electron paramagnetic resonance, have provided unprecedented insights into the role of surface defects in photocatalytic processes [193,194,195,196]. Table 2 outlines the major advancements in modifying TiO_2_ photocatalysts for visible light-driven hydrogen production, focusing on defect engineering, doping strategies, and surface modifications.

To provide a quantitative comparison of different TiO_2_-based photocatalysts and their hydrogen production efficiencies, Table 3 summarizes various modified TiO_2_ photocatalysts and their fabrication methods, testing conditions, and corresponding hydrogen evolution rates.

The analysis of surface modification strategies reveals several important considerations. Black TiO_2_, while showing impressive visible light absorption, often exhibits a decreased stability compared to pristine TiO_2_, with oxygen vacancies prone to healing under oxidizing conditions [83]. The trade-off between enhanced visible light absorption and long-term stability remains a significant challenge [83,225]. Co-doping strategies, though effective in band gap engineering, can introduce complex defect structures that may act as recombination centers, potentially negating the benefits of enhanced light absorption [226,227]. The optimal dopant concentration represents a delicate balance between an improved visible light absorption and maintained charge carrier mobility [228].

### 4.3. Advanced Heterojunction Systems and Z-Scheme Design

Heterojunction engineering has witnessed significant progress, particularly in Z-scheme systems, where the integration of 2D materials such as g-C_3_N_4_ and MXenes has established new paradigms for electron transfer [229,230,231,232,233]. Direct Z-scheme systems, eliminating the need for electron mediators, have demonstrated an unprecedented charge separation efficiency while maintaining a robust stability under prolonged operation [234,235,236]. The development of atomically sharp interfaces and the implementation of strain engineering at heterojunctions have led to significant improvements in charge transfer efficiency, with some systems approaching theoretical maximum quantum yields [237]. However, poor interface engineering can lead to charge accumulation and increased recombination rates [238]. The stability of these interfaces under operating conditions, particularly in Z-scheme systems, remains a crucial challenge [239,240]. Moreover, the complexity of multi-component systems can lead to increased manufacturing costs and a reduced scalability [241,242].

### 4.4. Cocatalyst Integration and Interface Optimization

Cocatalyst integration has evolved substantially, with single-atom catalysts and bimetallic systems showing an exceptional hydrogen evolution performance. The development of core–shell structures and protective layers has addressed long-standing stability challenges, while interface engineering has optimized charge transfer dynamics [243,244]. Recent advances in synthetic methods have enabled precise control over cocatalyst size, distribution, and electronic structure, leading to significant improvements in catalytic activity and stability [81,245]. 

Despite their advantages, cocatalyst systems face several critical challenges. Single-atom catalysts, while highly active, often suffer from aggregation during operation, leading to a decreased performance over time [246,247]. The high cost of noble metal cocatalysts remains a significant barrier to commercial implementation [248]. Interface optimization between cocatalysts and TiO_2_ surfaces requires precise control over surface chemistry and morphology, which can be challenging to maintain during scale-up [249,250,251].

### 4.5. Current Challenges and Future Perspectives

Despite remarkable advances in the nanoscale engineering of TiO_2_-based photocatalysts, several critical challenges impede widespread implementation. While hierarchical nanostructures have demonstrated enhanced light harvesting through an increased surface area and improved charge transport pathways, their complex synthesis often results in an inconsistent reproducibility between research batches [252]. The integration of ordered arrays and self-assembled structures, though promising for directional charge transport, faces significant scalability challenges, with production costs typically 5–10 times higher than those for conventional materials [26,253]. Crystal-phase engineering, particularly in mixed-phase TiO_2_ systems, namely anatase–rutile heterojunctions, has shown an enhanced charge separation efficiency, but often suffers from phase instability under prolonged operation, with anatase-to-rutile transformation accelerated by reaction conditions [254,255]. The introduction of surface defects and oxygen vacancies, while effective for visible light absorption, frequently leads to a reduced photocatalytic stability due to their metastable nature, with surface defects being particularly susceptible to repair through water and oxygen adsorption [256]. These limitations highlight the critical need for balanced material design approaches that consider not only performance metrics, but also the practical aspects of scalability, stability, and cost-effectiveness.

Future directions in advanced materials design point toward several promising avenues. The exploration of earth-abundant cocatalysts with a high activity and stability remains a priority, as does the investigation of dynamic interface phenomena under reaction conditions [257,258,259]. The integration of design principles across multiple time and length scales, coupled with advanced in situ characterization techniques, is expected to provide deeper insights into reaction mechanisms and degradation pathways [260,261]. Additionally, the development of scalable synthesis methods for complex nanostructures and the optimization of interface engineering strategies continue to be active areas of research [262,263]. This evolution in materials design represents a significant step toward the practical, large-scale implementation of photocatalytic water splitting systems. However, continued research is essential to address the remaining challenges in stability, efficiency, and cost-effectiveness. The integration of multiple design strategies, coupled with advanced characterization and theoretical modeling, provides a promising pathway toward achieving commercially viable photocatalytic water splitting systems [264,265]. 

## 5. Hydrogen Production from Different Water Sources: Effects and Applications

The development of efficient and sustainable photocatalytic water splitting systems requires the careful consideration of water source characteristics. While significant advancements have been made in photocatalytic technologies, the transition from laboratory conditions to real-world water sources presents distinct challenges. Different water sources, such as seawater, municipal wastewater, and industrial wastewater, introduce unique complexities that affect both the performance and stability of TiO_2_-based photocatalysts. Understanding these effects and developing appropriate solutions is crucial for practical applications. 

### 5.1. Necessity and Feasibility Analysis of Alternative Water Sources

The increasing global demand for clean hydrogen production necessitates exploring alternative water sources beyond purified water from freshwater supplies, driven by environmental, economic, and sustainability considerations [266]. Freshwater accounts for only 2.5% of the global water resources, with much of it being inaccessible [267]. Industrial-scale hydrogen production using pure water could potentially stress already limited freshwater resources, particularly in water-scarce regions [268]. Economic analysis reveals that advancements in photocatalytic hydrogen production, including seawater-based systems, have the potential to significantly reduce operational costs compared to conventional freshwater-based approaches [26]. Integration with wastewater treatment offers dual economic benefits through simultaneous pollutant degradation and hydrogen generation [34]. Furthermore, utilizing alternative water sources for hydrogen production, including seawater-based processes, presents environmental benefits such as a reduced dependency on freshwater in water-stressed regions. Additionally, integrating renewable energy with hydrogen production can lead to significant reductions in greenhouse gas emissions, though the specific carbon footprint reduction varies depending on process efficiency and energy input [269,270]. 

Among these alternative water sources, seawater emerges as a particularly promising candidate, constituting 96.5% of the Earth’s water supply. Its vast availability and proximity to coastal regions with high energy demands make it an attractive sustainable alternative to freshwater resources [271]. However, significant challenges exist in seawater hydrogen production, including the need for complex pretreatment processes to remove impurities and manage chloride ions, which can accelerate catalyst degradation and impede system stability [272,273]. Moreover, the presence of inorganic salts such as Mg^2+^ and Na^+^ in seawater may affect long-term photocatalytic hydrogen production by potentially deactivating catalysts and altering their physical and chemical properties. These dissolved salts can reduce hydrogen evolution rates by consuming photogenerated carriers, which may require strategies to mitigate their impact on catalyst performance [271]. 

While seawater offers abundant supply, municipal wastewater presents unique opportunities for photocatalytic hydrogen production by utilizing organic pollutants as sacrificial electron donors, enabling simultaneous pollutant removal and hydrogen generation. This approach aligns with sustainable wastewater treatment strategies, offering potential integration with existing treatment infrastructure for enhanced energy recovery [274,275,276]. This integration can substantially reduce treatment costs through a dual functionality. However, the variable composition of municipal wastewater affects the process stability in photocatalytic hydrogen production, as different contaminants influence catalyst performance. Additionally, biofilm accumulation and reactor fouling present significant operational challenges. The presence of organic inhibitors and heavy metals necessitates advanced monitoring and careful system design to prevent catalyst deactivation and ensure stable hydrogen production [277]. 

Beyond municipal sources, industrial wastewater systems, such as those from the paper and pulp industry, contain high concentrations of organic electron donors that can be utilized for photocatalytic hydrogen production. This process offers the dual benefits of energy generation and pollutant removal, demonstrating the potential for industry-specific optimization [276,278]. The integration of photocatalytic hydrogen production with industrial wastewater treatment provides dual benefits by enabling simultaneous pollutant removal and energy recovery. However, these systems face challenges due to the presence of toxic compounds, including heavy metals and refractory organic pollutants, which necessitate complex pretreatment strategies. Additionally, variations in pH significantly influence photocatalytic efficiency and can lead to catalyst instability, requiring careful operational control [276,278,279].

The successful implementation of these alternative sources hinges critically on understanding their chemical characteristics and how they influence catalyst performance, which leads directly to the examination of water chemistry’s role in photocatalytic efficiency, which is discussed in the following section.

### 5.2. Influence of Water Chemistry on Photocatalytic Performance

The efficiency of TiO_2_-based photocatalytic water splitting is profoundly influenced by water chemistry, which plays a crucial role in determining both reaction kinetics through pH-dependent charge distribution and ionic interactions, as well as catalyst stability [280,281]. Some investigations have revealed that pH, ionic strength, and dissolved species significantly impact the surface chemistry of TiO_2_ and the kinetics of water splitting reactions [69,282,283,284,285,286,287]. Modulating pH not only affects the band edge positions of TiO_2_, but also influences the surface charge distribution, which, in turn, alters the adsorption–desorption equilibrium of reactive species like water and protons [288,289]. The presence of common ions such as Na^+^, Cl^-^, SO_4_^2-^, etc., in natural water sources modifies the local electric field at the semiconductor/electrolyte interface, affecting charge carrier separation and transport efficiency [290,291]. In addition, dissolved organic matter (DOM) can serve as both electron donors and acceptors, introducing competing reaction pathways that may either enhance or inhibit hydrogen evolution rates [292,293]. Specifically, while some ions (e.g., sulfate ions) stabilize charge carriers and enhance photocatalytic activity, others (e.g., chloride ions) may cause catalyst degradation [294,295,296]. Moreover, dissolved oxygen can act as an electron acceptor, competing with hydrogen evolution, thus reducing the overall photocatalytic performance [297]. The intricate interplay of these water chemistry factors underscores the complexity of optimizing TiO_2_-based photocatalysts for practical hydrogen production. The key water chemistry parameters and their specific effects on the photocatalytic performance in TiO_2_-based systems are summarized in Table 4.

### 5.3. Impact of Dissolved Species and Impurities

The presence of dissolved species and impurities in water presents both challenges and opportunities for photocatalytic water splitting. Certain ionic species such as Ca^2+^, Zn^2+^, Fe^2+^, and Fe^3+^ can adversely affect catalyst performance by causing surface poisoning through competitive adsorption or the formation of inactive surface complexes, as observed with multivalent metal ions that induce surface precipitation and alter the electric double-layer structure [299,300]. These interactions can reduce the availability of active sites and disrupt charge carrier dynamics, thereby diminishing overall efficiency [301]. Conversely, some dissolved species such as triethylamine and potassium iodide offer advantageous effects, acting as hole scavengers or facilitating charge separation by forming beneficial surface complexes [302,303,304]. 

### 5.4. Advanced Strategies for Seawater Splitting

Seawater splitting offers a sustainable pathway for large-scale hydrogen production yet presents distinct challenges due to its complex chemical composition and high salinity. The primary issues include chloride-induced catalyst degradation, fouling, and scaling through the formation of Ca(OH)_2_ and Mg(OH)_2_ deposits [305,306,307]. To address these challenges, recent advancements have focused on developing chloride-resistant photocatalysts through surface modifications and protective layer integration [308,309,310]. Strategies such as selective membranes and buffer layers have been shown to mitigate the corrosive effects of chloride ions while preserving a high photocatalytic activity [311,312]. Additionally, the incorporation of engineered cocatalyst systems has demonstrated the ability to sustain stability in high-salt environments, achieving hydrogen evolution rates comparable to those observed in pure water systems [313,314]. The performance metrics of various TiO_2_-based photocatalysts designed for seawater splitting, along with their corresponding strategies, are presented in Table 5.

### 5.5. Integration with Wastewater Treatment Systems

The integration of photocatalytic hydrogen production with wastewater treatment offers a transformative approach to concurrently addressing energy and environmental challenges. TiO_2_-based photocatalysts have demonstrated a promising potential for simultaneous hydrogen evolution and the degradation of organic pollutants [34,322,323]. Advanced reactor configurations, including membrane separation and continuous flow systems, have been developed to maintain a stable hydrogen production while achieving substantial reductions in organic contaminant concentrations [324,325]. Organic pollutants present in wastewater effectively serve as sacrificial electron donors, thereby enhancing hydrogen evolution rates and enabling efficient water purification [326]. Despite these advancements, key challenges, such as catalyst deactivation and ensuring long-term stability within the chemically complex matrices of wastewater, remain [327,328]. Future research should focus on optimizing catalyst designs and reactor configurations to address these limitations and enhance the scalability of these integrated systems. To illustrate the current state of technology, Table 6 presents the simultaneous hydrogen production and wastewater treatment performance of selected TiO_2_-based catalysts.

### 5.6. Future Prospectives and Challenges

The translation of laboratory-scale success to real-world water sources reveals significant performance gaps in TiO_2_-based photocatalytic systems. While impressive results have been achieved in controlled laboratory conditions, performance typically decreases when applied to actual environmental waters [82]. Common ionic species present in natural waters, particularly chloride and carbonate ions, can significantly reduce photocatalytic efficiency through competitive adsorption and radical scavenging effects [341,342]. The deposition of carbonaceous compounds on the catalyst surface, known as fouling or coking, is a common cause of deactivation that can block active sites and pores, often necessitating frequent regeneration cycles, which impact the long-term operational costs and viability of the process [343]. Moreover, the synergistic effects of multiple ions and organic compounds in real water sources create complex interaction patterns that current theoretical models fail to fully predict [344]. Standard testing protocols often overlook these crucial matrix effects, leading to overoptimistic performance projections that rarely translate to practical applications [345]. This disconnect between idealized laboratory conditions and real-world performance necessitates a fundamental shift in how we evaluate and design photocatalytic systems for practical water splitting applications [346].

Despite notable advancements, significant challenges remain in the practical implementation of photocatalytic water splitting systems, particularly when using non-traditional water sources such as seawater or wastewater. One critical issue is ensuring long-term stability under variable and often harsh water chemistry conditions [347]. The development of robust and scalable catalyst systems capable of maintaining activity and selectivity in such environments is imperative [348]. Furthermore, optimizing integrated treatment processes to handle complex water matrices without compromising hydrogen production efficiency is essential for widespread application [349].

Future research should prioritize the development of smart, self-healing materials that can adapt dynamically to fluctuating water chemistry, such as changes in pH, ionic strength, or contaminant levels [350]. Advancements in in situ and operando characterization techniques are needed to provide real-time insights into reaction mechanisms and surface interactions, which are crucial for understanding and mitigating deactivation pathways [260,261,347]. Additionally, the establishment of standardized protocols for performance evaluation under realistic operating conditions is necessary to facilitate reliable comparisons and accelerate the translation of laboratory-scale findings to industrial applications [351]. The integration of artificial intelligence (AI) and machine learning approaches holds immense potential in optimizing system performance. These technologies can be leveraged to model complex reaction networks, predict catalyst behavior across diverse water sources, and design adaptive operational strategies [349,351]. By combining AI-driven insights with experimental advancements, researchers can streamline the development of next-generation water splitting systems [350].

## 6. Scale-Up and Engineering Challenges

### 6.1. Reactor Design Considerations and Optimization

The scaling of photocatalytic water splitting systems from the laboratory to the industrial scale presents significant engineering challenges that demand innovative reactor design solutions. Recent advances in reactor engineering have focused on optimizing light utilization efficiency, mass transfer, and reaction kinetics while maintaining economic viability [264,352,353]. Advanced reactor configurations, including suspended particle systems, fixed-bed reactors, and optical fiber reactors, have demonstrated varying degrees of success in addressing scale-up challenges [354]. Computational fluid dynamics modeling has emerged as a powerful tool for predicting flow patterns, light distribution, and reaction kinetics in large-scale reactors, enabling more efficient design optimization [355]. Furthermore, modular reactor systems have shown promise in addressing scalability issues, offering operational flexibility and ease of maintenance, which are critical for real-world applications [356]. The key characteristics, challenges, and engineering solutions for major photocatalytic reactor designs are summarized in Table 7.

### 6.2. Light Distribution and Mass Transfer Phenomena

The optimization of light distribution represents a critical challenge in large-scale photocatalytic systems. Non-uniform light distribution in scaled-up reactors has been shown to significantly affect the overall system efficiency, leading to a suboptimal photocatalytic performance [28,353]. Advanced light delivery systems, including internal illumination configurations and solar concentrators, have been developed to enhance the light utilization efficiency in photocatalytic reactors [360,361]. Additionally, the integration of plasmonic materials and photonic crystals has demonstrated significant improvements in light harvesting and uniform light distribution throughout the reactor volume [362,363,364,365,366]. Addressing mass transfer limitations, particularly in gas–liquid–solid systems, remains a key focus of reactor design. Innovative solutions, such as enhanced mixing strategies and optimized flow patterns, have been implemented to improve mass transfer dynamics [367,368,369]. The incorporation of structured catalysts and membrane-integrated systems has further demonstrated enhanced mass transfer characteristics, ensuring a high photocatalytic activity under operational conditions [370,371]. The major technical challenges encountered in large-scale photocatalytic reactor design and their corresponding solutions are presented in Table 8.

While various strategies have been proposed to address these challenges, a quantitative assessment of the key scalability factors is essential to guide future advancements in photocatalytic hydrogen production. Table 9 summarizes the key engineering challenges in scaling up photocatalytic hydrogen production, providing quantitative data on efficiency losses, material limitations, and proposed solutions derived from recent advancements.

### 6.3. Process Integration and System Optimization

The successful implementation of large-scale photocatalytic water splitting systems requires the careful consideration of process integration and system optimization. Recent developments have focused on integrating hydrogen separation and purification systems, thermal management strategies, and control systems to achieve an optimal performance [310,384]. Incorporating renewable energy sources, such as solar and wind power, for supplementary energy needs has emerged as a promising approach to enhance the sustainability of these systems. Hybrid systems that combine photocatalytic and conventional water splitting techniques have demonstrated an improved overall system efficiency and adaptability under varying operational conditions [385]. 

Advanced process control strategies leveraging machine learning algorithms and real-time monitoring systems have been instrumental in enhancing system performance and reliability. These technologies enable the prediction and adjustment of operational parameters to maintain a stable performance under fluctuating conditions [386]. Furthermore, the integration of heat recovery systems has been shown to improve energy efficiency by capturing and reusing the waste heat generated during the process [387]. 

### 6.4. Economic Feasibility and Sustainability Analysis

Economic considerations play a pivotal role in determining the commercial viability of scaled-up photocatalytic water splitting systems. Recent techno-economic analyses have highlighted key cost drivers and potential strategies for optimization, including advancements in reactor construction, catalyst synthesis, and the development of auxiliary equipment [388,389]. Capital costs, particularly those related to the production and scalability of photocatalysts, remain a significant barrier to widespread adoption, while operating costs, encompassing maintenance, catalyst replacement, and energy consumption, have been systematically evaluated to identify areas for cost reduction and efficiency improvements [390,391]. Life cycle assessment studies have underscored the importance of incorporating environmental and sustainability metrics into system design and optimization [392]. These assessments provide insights into the long-term environmental impacts, such as carbon emissions and resource utilization, associated with large-scale photocatalytic hydrogen production. Furthermore, the development of cost-effective methods for catalyst production, including scalable and sustainable synthesis techniques, has emerged as a critical area of research [393,394]. In addition, the integration of renewable energy sources for supplementary power, combined with strategies to minimize waste and recycle key materials, has shown promise in enhancing the economic feasibility and sustainability of these systems [34]. By addressing these economic and environmental challenges, future developments can pave the way for the commercialization of photocatalytic hydrogen production technologies.

### 6.5. Future Direction and Research Needs

Pilot and full-scale TiO_2_-based photocatalytic systems have revealed fundamental limitations that are often masked in bench-scale studies. While bench-scale reactors demonstrate promising efficiencies, scaling to industrial volumes results in significant performance losses, primarily due to light distribution limitations and mass transfer constraints [355,395]. Current reactor designs struggle to maintain a uniform light intensity throughout larger volumes, with light penetration depth rarely exceeding 5 cm in typical slurry systems [372,396]. While advanced light delivery systems like optical fibers and solar concentrators offer potential improvements, they present significant engineering challenges in maintaining uniform light distribution and thermal management [397,398]. Mass transfer limitations are a major challenge in scaled-up systems, with a recent survey showing that 30% of scale-up failures were attributed to mass transfer issues and reaction times could potentially increase 4–5 fold without proper controls [399]. The economic viability of large-scale implementation remains questionable, with techno-economic analyses indicating photocatalyst costs need to be below USD 10/kg and solar-to-hydrogen efficiencies above 10% for commercial viability—targets that remain elusive with existing technologies [242].

Future advancements in the scaling up and engineering of photocatalytic water splitting systems must address several crucial areas to ensure both efficiency and commercial viability. Key research directions include the following:

▪ The development of advanced reactor designs that integrate improved light delivery systems and enhanced mass transfer characteristics, aiming to maximize the system’s overall performance.
▪ The incorporation of artificial intelligence and machine learning approaches for the optimization of operational strategies, system control, and real-time monitoring [400].
▪ The implementation of sustainable and cost-effective manufacturing processes for catalyst production and system components, ensuring scalability and resource efficiency.
▪ The establishment of standardized methodologies for performance evaluation, including techno-economic and life cycle assessments, to streamline industry adoption and regulatory compliance.

Furthermore, addressing the challenges related to catalyst recovery and regeneration, system maintenance, and ensuring long-term stability under industrial conditions is essential for the practical application of photocatalytic water splitting technologies [401,402].

## 7. Advanced Applications and System Integration of TiO_2_-Based Photocatalysts

The advanced integration of TiO_2_-based photocatalysts encompasses multiple strategic approaches, including bio-hybrid systems, CO_2_ reduction, and machine learning applications. These integration strategies present both opportunities and challenges in advancing photocatalytic technology. 

### 7.1. Integration with Artificial Photosynthesis

The integration of photocatalytic water splitting with artificial photosynthesis offers a promising path for advancing sustainable energy solutions. Recent progress has demonstrated the successful incorporation of photocatalysts with synthetic biological components, resulting in an enhanced energy conversion efficiency [403]. Novel bio-hybrid systems, which integrate engineered proteins and light-harvesting complexes, have shown significant improvements in solar-to-hydrogen conversion rates, outperforming traditional systems [404]. These hybrid systems combine the selectivity of biological catalysts with the robustness and stability of inorganic semiconductor materials, making them ideal candidates for large-scale energy production [405,406]. Moreover, advanced architectures that combine multiple photocatalytic centers with biomimetic electron transfer pathways have achieved a remarkable stability and improved quantum efficiency under visible light irradiation, thereby enhancing overall system performance and longevity [407,408]. The major innovations in bio-hybrid photocatalytic systems and their corresponding significance in advancing sustainable energy solutions are detailed in Table 10.

While bio-hybrid photocatalytic systems present an attractive pathway for mimicking natural photosynthesis, significant technical barriers impede their practical implementation. The integration of biological components with inorganic photocatalysts, though promising in principle, faces considerable challenges, including a limited operational stability, low energy conversion efficiency, and poor sustainability of the catalytic system during continuous operation [414,415]. Current bio-hybrid photocatalytic systems for water splitting hydrogen production still face efficiency challenges. Although these systems show promise, their hydrogen evolution rates need further improvement for practical applications [407,416]. The structural complexity and synthesis requirements of these bio-hybrid systems currently present challenges for large-scale production and practical implementation. While these systems show a promising potential in mimicking natural photosynthesis and offer unique advantages in biomass conversion, their widespread adoption is limited by the need for the following:

▪ Improved photocatalytic efficiency and stability
▪ Enhanced charge separation and transfer
▪ Optimization of light harvesting capabilities
▪ Development of more cost-effective alternatives to noble metal cocatalysts

These limitations suggest that, while bio-hybrid systems represent an innovative approach, continued advances in materials design, synthesis methods, and interface engineering are necessary to make them commercially competitive with conventional photocatalysts [417,418].

### 7.2. Coupling with CO_2_ Reduction Systems

The integration of hydrogen production with CO_2_ reduction in photocatalytic systems represents a transformative approach to sustainable energy and chemical synthesis. Recent advancements have centered on the development of dual-function catalysts capable of simultaneously facilitating water oxidation and CO_2_ reduction [419]. The incorporation of selective cocatalysts, such as metal oxides and molecular complexes, has significantly improved reaction efficiency and product selectivity [420]. Z-scheme photocatalytic systems, which mimic natural photosynthesis, have demonstrated the ability to couple water splitting and CO_2_ reduction with an enhanced energy conversion efficiency [421]. Emerging approaches leveraging plasmonic effects and defect engineering have shown great promise in enhancing CO_2_ activation while maintaining a robust water splitting performance [422,423]. 

### 7.3. Machine Learning Applications in Materials Discovery

The application of machine learning has significantly advanced the discovery and optimization of photocatalytic materials, enabling the efficient identification of promising candidates and pathways. Advanced machine learning algorithms have been instrumental in rapidly screening vast material combinations and accurately predicting photocatalytic performance based on theoretical and experimental data [71,424]. Deep learning models have revealed intricate structure–property relationships, aiding in the prediction of optimal synthesis conditions for novel photocatalysts [425]. 

The integration of high-throughput experimentation with machine learning-driven analysis has accelerated the pace of material discovery. These combined approaches allow researchers to evaluate numerous variables simultaneously, reducing the time and resources required for experimental validation [426,427,428]. Notably, interpretable machine learning models have emerged as a valuable tool, offering insights into fundamental mechanisms governing photocatalytic activity, stability, and efficiency. These models provide transparent and explainable predictions, bridging the gap between computational predictions and experimental realities [71,429,430,431]. 

## 8. Hydrogen Storage Considerations in Photocatalytic Systems

Photocatalytic water splitting is a promising approach for sustainable hydrogen generation. However, fluctuations in solar irradiation lead to variable hydrogen production rates, necessitating effective storage solutions such as strategic hydrogen reserves to mitigate seasonal mismatches and ensure stable supply [432]. The choice of storage technology significantly impacts overall system efficiency and the feasibility of large-scale implementation. 

For photocatalytic applications, chemical storage methods, particularly metal hydrides and liquid organic hydrogen carriers (LOHCs), provide advantages such as reversible hydrogenation/dehydrogenation, long-term storage stability, and compatibility with existing energy infrastructure, making them superior to conventional physical storage approaches [433,434,435]. Metal hydrides, such as TiFeH_2_, MgH_2_, and LaNi_5_H_6_, enable efficient hydrogen storage at moderate pressures (1–10 bar), a range commonly achievable in photocatalytic reactors. These materials reduce the need for energy-intensive compression while maintaining stable and reversible hydrogen absorption and desorption properties [436,437,438]. However, their widespread application is constrained by challenges related to hydrogen sorption kinetics and thermal management, as inefficient heat dissipation during absorption–desorption can lead to thermal instability and a reduced cycle life, necessitating further optimization [439,440,441]. LOHCs, such as N-ethylcarbazole and perhydro-dibenzyltoluene, enable ambient-condition hydrogen storage and can be integrated with catalytic hydrogenation units to facilitate efficient hydrogen storage and release. Their compatibility with existing infrastructure makes them a viable solution for distributed hydrogen production and transport [442,443]. In contrast, physical hydrogen storage methods, including compression at 700 bar and liquefaction at −253 °C, impose considerable energy penalties. Compression requires at least 4.1 wt% energy input, with additional cooling energy demands of 1.8–3.6 wt%, while liquefaction processes have an efficiency of around 70% and require significant infrastructure investments. These factors diminish the efficiency advantages of photocatalytic hydrogen production [433]. Cryo-compressed hydrogen storage (250–350 bar, 20–80 K) has emerged as a promising alternative, offering the highest system storage densities while effectively reducing vent losses compared to conventional cryogenic storage. However, system complexity, insulation performance, and thermodynamic drift remain key challenges requiring further optimization [444]. 

The development of integrated photocatalytic storage systems remains an active research area, with ongoing efforts focused on optimizing storage compatibility with fluctuating pressure profiles in photocatalytic hydrogen production while ensuring safety, efficiency, and cost-effectiveness [390,445]. Future innovations should focus on minimizing energy losses in hydrogen storage while maintaining practical energy densities, ensuring stable operation under fluctuating pressure conditions. Additionally, advancements should enable seamless integration with renewable energy grids and hydrogen-based infrastructure to enhance efficiency, safety, and cost-effectiveness.

## 9. Future Perspectives and Emerging Opportunities in Photocatalytic Water Splitting

The field of photocatalytic water splitting stands at a critical juncture, where, despite significant scientific advances, practical implementation remains elusive. Current state-of-the-art systems achieve solar-to-hydrogen efficiencies of only 1–2% under real-world conditions, far below the 10% threshold considered necessary for commercial viability [391,446]. The cost of hydrogen production through photocatalytic water splitting remains 3–4 times higher than conventional methods, primarily due to expensive materials and complex system requirements [26,390]. Stability issues remain a critical challenge in photocatalytic devices, with the most advanced systems demonstrating limited operational lifetimes. Current research indicates that, while a five-year lifetime (approximately 21,900 h) is required for cost-competitive hydrogen production, most cutting-edge systems struggle to maintain a performance beyond 100 h [447]. These fundamental challenges suggest that revolutionary rather than evolutionary advances are needed across multiple fronts—from basic materials design to system engineering [448]. The field must address not only efficiency and stability, but also scalability and economic viability to bridge the gap between laboratory success and commercial implementation [449]. Promising areas for future exploration include the following:

▪ Efficient Visible-Light-Responsive Materials: Advanced bandgap engineering has enabled the design of photocatalysts capable of harnessing a broader spectrum of sunlight, significantly enhancing overall efficiency [450,451].
▪ Quantum Computing for Materials Optimization: Quantum computing approaches are being explored for the accelerated discovery and optimization of photocatalytic materials, offering potential breakthroughs in reaction efficiency and material design [452].
▪ Autonomous Systems for Real-Time Optimization: Autonomous systems equipped with real-time monitoring and adaptive control mechanisms are transforming operational efficiency and system reliability [453].
▪ Sustainable Manufacturing Processes: A focus on the eco-friendly manufacturing of advanced photocatalytic materials ensures scalability and minimizes environmental impact [454].
▪ Smart Photocatalytic Systems: Systems capable of adapting to environmental changes (e.g., a varying water quality or solar intensity) have the potential for robust operation under diverse conditions [26,455].

Addressing challenges related to long-term stability, scalability, and cost-effectiveness is essential for commercial success. Recent trends also highlight the development of multifunctional systems capable of tackling multiple environmental issues concurrently, such as simultaneous water purification and hydrogen production [26,449].

**Figure 4:**
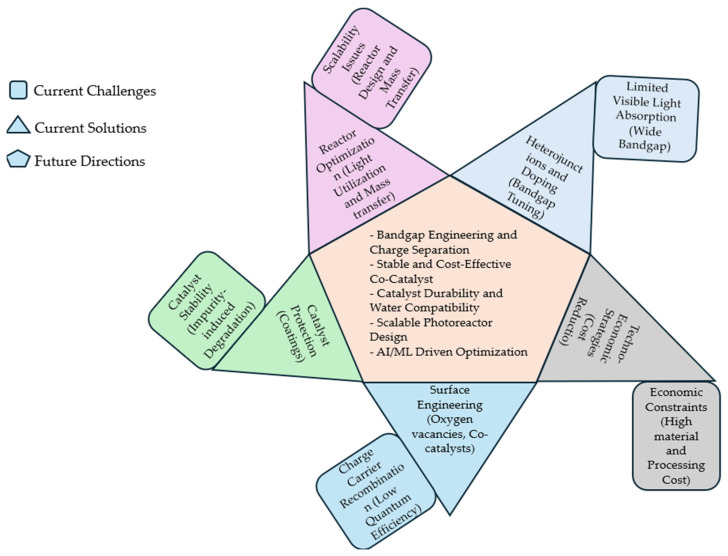
TiO_2_-based photocatalysis: from challenges to implementation.

In conclusion, while significant strides have been made in photocatalytic water splitting, achieving commercial viability demands transformative innovations that address critical challenges such as efficiency, stability, scalability, and cost-effectiveness (Figure 4). By advancing material design, leveraging emerging technologies like quantum computing and autonomous systems, and prioritizing sustainable manufacturing, the field can move closer to bridging the gap between laboratory success and real-world implementation. A multidisciplinary approach that integrates these solutions will be pivotal in unlocking the full potential of photocatalytic water splitting as a sustainable pathway for green hydrogen production.

## Figures and Tables

**Table 1:** Key advancements in TiO_2_-based photocatalysts.

Advancement	Key Features	Impact on Efficiency	Reference
Hierarchical Nanostructures	Integration of nanotubes, nanosheets, and nanoparticles into cohesive architectures.	Enhanced light scattering and trapping mechanisms, leading to improved photocatalytic hydrogen production.	[171]
Macro/Mesoporous Structures	Engineered pores facilitating superior mass transport and maximizing reactive surface area.	Significant improvements in photocatalytic hydrogen production due to enhanced surface area and reaction kinetics.	[172]
Ordered Arrays and Self-Assembly	Formation of ordered arrays and self-assembled structures for directional charge transport.	Addressed limitations in traditional designs by enhancing charge separation and reducing recombination rates, leading to improved hydrogen evolution.	[173]
Crystal-Phase Engineering	Controlled synthesis of mixed-phase TiO_2_ (anatase/rutile) junctions.	Exceptional charge separation properties, significantly reducing recombination rates and enhancing hydrogen production efficiency.	[174]
Nanoscale Engineering	Precise control over TiO_2_ nanostructures, including size, shape, and surface properties.	Enhanced light absorption, charge separation, and surface reaction kinetics, leading to improved hydrogen production efficiency.	[175]

**Table 2:** Advances in surface chemistry and defect engineering.

Advancement	Key Features	Impact on Efficiency	Reference
Black TiO_2_ via Oxygen Vacancy Engineering	Controlled introduction of oxygen vacancies to create black TiO_2_ with enhanced visible light absorption.	Extends photocatalytic activity into the visible spectrum, significantly improving hydrogen production rates.	[184]
Surface Modification and Defect Engineering	Atomic-level control over surface defects, including Ti^3+^ states and non-metal doping.	Improves charge separation and extends light absorption, leading to higher hydrogen evolution rates.	[138]
Co-doping with Transition Metals and Non-Metals	Incorporation of both metal and non-metal dopants into TiO_2_ lattice.	Synergistic effect leading to enhanced visible light absorption and improved hydrogen production rates.	[197]

**Table 3:** Summary of modified TiO_2_-based photocatalysts and hydrogen production efficiencies.

Photocatalyst Composition	Fabrication Method	Hydrogen Production Rate	Light Source	Reference
Ag/TiO_2_	Chemical Reduction	23.5 mmol g^−1^ h^−1^	UV lamp (254 nm Wavelength)	[198]
Co_3_O_4_@C/TiO_2_	Carbonization	11,400 µmol g^−1^ h^−1^	UV-LED lamp (365 nm Wavelength)	[199]
CuO/TiO_2_	Hydrothermal	2000 µmol g^−1^ h^−1^	300 W Xe lamp	[200]
Co_3_O_4_ QDs/TiO_2_	Hydrothermal	1735 µmol g^−1^ h^−1^	Solar light	[201]
Co3O4@TiO_2_/Pt	Hydrothermal	5280 µmol g^−1^ h^−1^	300 W Xe lamp	[202]
CoOx-TiO_2_/CdS	Solvothermal	660 µmol g^−1^ h^−1^	Visible light (> 400 nm Wavelength)	[203]
Pt/TiO_2_	Hydrothermal	334 µmol h^−1^	350 W Xe lamp	[172]
FP-Pt/TiO_2_	Pyrolysis	19.25 mmol g^−1^ h^−1^	300 W Xe lamp	[204]
FP-Cu/TiO_2_	Pyrolysis	5.02 mmol g^−1^ h^−1^	300 W Xenon lamp	[204]
Cr-TiO_2_	Magnetron sputtering and Sol–gel	5.3 µmol h^−1^	Visible light	[205]
Fe-TiO_2_	Magnetron sputtering and Sol–gel	15.5 µmol h^−1^	Visible light	[205]
Cu-TiO_2_ (P25)	Photoassisted Deposition	8.47 mmol g^−1^ h^−1^	450 W Hg lamp	[206]
Co-TiO_2_	Photoassisted Deposition	2.48 mmol g^−1^ h^−1^	450 W Hg lamp	[206]
Fe-TiO_2_	Microwave-Hydrothermal	11 µmol g^−1^ h^−1^	Xe lamp	[207]
Fe-TiO_2_	Impregnation	230 µmol g^−1^ h^−1^	UV light	[208]
Ru-TiO_2_	Micro-emulsion	0.80 mmol g^−1^ h^−1^	500 W Xe lamp	[209]
Au-TiO_2_	Photodeposition	1.1 mmol g^−1^ h^−1^	UV lamp	[210]
Pd/N-TiO_2_	Chemical vapor deposition	6.3 mmol g^−1^ h^−1^	White LED	[211]
Fe-Ni-/Ag/TiO_2_	Solvothermal	794 µmol g^−1^ h^−1^	500 W Xe lamp	[212]
Pt/Mg-TiO_2_	Hydrothermal	850 µmol g^−1^ h^−1^	300 W Xe lamp	[213]
Pt SA/Def-s-TiO_2_	Deposition–Precipitation	13.5 mmol g^−1^ h^−1^	300 W Xe lamp	[214]
Cu-TiO_2_	Ball Milling	9.5 mmol g^−1^ h^−1^	300 W Xe lamp	[215]
Ni-TiO_2_	Molten Salt	1.9 mmol g^−1^ h^−1^	300 W Xe lamp	[216]
Sn/TiO_2_	Photoinduced Deposition	553 µmol g^−1^ h^−1^	3 W UV lamp	[217]
N-TiO_2_	Sol–gel and Electrospinning	28 µmol h^−1^	150 W Xe lamp	[218]
N-TiO_2_	RF Magnetron Sputtering Deposition	4.5 mmol cm^−2^ h^−1^	300 W Xe lamp	[219]
N-TiO_2_ with VO	Solvothermal	1.04 mmol g^−1^ h^−1^	Solar Simulator	[220]
S-TiO_2_	Thermal Protection	164 µmol g^−1^ h^−1^	Visible light	[221]
TiC@C-TiO_2_	Situ Thermal Growth	558 µmol g^−1^ h^−1^	300 W Xe lamp	[222]
N/F-TiO_2_	Calcination	11.5 µmol g^−1^ h^−1^	300 W Xe lamp	[223]
C/N self-doped TiO_2_	Hydrothermal	332.3 µmol g^−1^ h^−1^	300 W Xe lamp	[188]
Br/N-TiO_2_	Hydrothermal	2.3 mmol g^−1^ h^−1^	300 W Xe lamp	[224]

**Table 4:** Water chemistry influence.

Factors	Effect of Photocatalytic Performance	References
pH	Modulates band edge positions, surface charge, and adsorption–desorption equilibrium of reactive species. Affects hydrogen evolution rates depending on the pH range.	[288,289]
Ionic Strength	Influences the electric double layer and charge carrier separation. High ionic strength can improve conductivity but may increase recombination.	[68,298]
DOM	Acts as electron donors and acceptors; competing pathways can either enhance or inhibit hydrogen evolution rates.	[292]
Common Ions (e.g., Na^+^, Cl^−^, SO_4_^2−^)	Alters the electric field at the semiconductor/electrolyte interface and influences charge carrier separation. Some ions stabilize the catalyst, while others lead to degradation.	[294,295]
Dissolved Oxygen	Competes with hydrogen evolution by capturing electrons, reducing overall photocatalytic efficiency.	[297]

**Table 5:** Strategies employed in seawater splitting.

Photocatalyst	Hydrogen Production Rate	Key Strategy/Condition Addressed	References
Mesoporous brookite/anatase TiO_2_	6.59 mmol g^−1^ h^−1^	Mitigated chloride-induced degradation	[315]
Brookite TiO_2_	1476 µmol g^−1^ h^−1^	Improved stability in saline environments	[305]
Phosphorus and Nickel co-doped TiO_2_	149 µmol g^−1^ h^−1^	Improved stability by preventing photo-corrosion	[316]
TiO_2_(NT)/Pt/Cd_0.8_Zn_0.2_S	21.7 mmol g^−1^ h^−1^	Increased active sites and hydrogen production rate	[317]
Granular Pt/TiO_2_	23.6 µmol h^−1^	Catalyst deflocculation and oxygen inhibition	[318]
WS_2_/C-TiO_2_/g-C_3_N_4_	986 µmol g^−1^ h^−1^	Enhanced electron–hole pair separation	[319]
MoS_2_@TiO_2_	580 mmol g^−1^ h^−1^	Enhanced plasmonic effect	[320]
SiO_2_/Ag@TiO_2_	816 µmol g^−1^ h^−1^	Photothermic interfacial heating synergy	[321]

**Table 6:** Simultaneous wastewater treatment and hydrogen production.

Photocatalyst	Hydrogen Production Rate	Pollutant Treated	Light Source	Reference
CuO/TiO_2_ NT	3.43 µmol g^−1^ h^−1^	Phenol	Visible light	[329]
Ag-G-TiO_2_	191 µmol g^−1^ h^−1^	Methylene Blue	Visible light	[34]
BiVO_4_/TiO_2_	14.3 mmol g^−1^ h^−1^	Rhodamine B	Visible light	[330]
NiPc@GO/TiO_2_	1.38 mmol h^−1^	Formic Acid	Visible light	[331]
7CuO-TiNTA	910 mmol/m^2^	Ammonia	UV light	[332]
Ag/TiO_2_	1729 µmol g^−1^ h^−1^	Paracetamol	Natural sunlight	[333]
MoS_2−x_ @TiO_2_-OV	42 µmol g^−1^ h^−1^	Pharmaceutical Wastewater	300 W Xenon lamp	[334]
MoS_2−x_ @TiO_2_-OV	103 µmol g^−1^ h^−1^	Coking wastewater	300 W Xenon lamp	[334]
PtCo_3_O_4_TiO_2_	2200 µmol g^−1^ h^−1^	Enrofloxacin	300 W Xenon lamp	[274]
Fe-doped TiO_2_	2423 µmol h^−1^	Methyl Orange	Visible light	[335]
Mixed TiO_2_ nanosphere and nanosheet	19.4 µmol g^−1^ h^−1^	Glycerol	300 W Xenon arc lamp	[336]
Pt/TiO_2_	101 µmol g^−1^ h^−1^	Oxalic Acid	450 W Xenon Arc lamp	[337]
Carbon-doped TiO_2_	374 µmolg^−1^h^−1^	Lactic Acid	Visible light	[338]
Cr_2_O_3_/Rh/SrTiO_3_	590 µmol g^−1^ h^−1^	4-chlorophenol	300 W Xe Arc lamp	[339]
Nanostructure mesoporous TiO_2_	19 mmol h^−1^	Olive mill wastewater	UV light	[340]

**Table 7:** Overview of photocatalytic reactor designs and scale-up challenges.

Reactor Type	Key Features	Scalability Challenges	Engineering Solutions	References
Suspended Particle	High light absorption, dynamic particle flow	Potential clogging, complex design	Optimization of particle dispersion and flow dynamics	[357]
Fixed-Bed Reactor	Stable, large-scale suitability	Low light utilization, heat management	Reactor designed for improved light penetration and mass transfer	[358]
Optical Fiber Reactor	Efficient light delivery through fibers, compact	Cost, complexity in large-scale integration	Optimization of fiber configuration for large-scale light distribution	[359]

**Table 8:** Key technical challenges and solutions in large-scale photocatalytic reactor design.

Aspect	Challenges	Solutions	References
Light Distribution	- Non-uniform illumination in scaled reactors leading to efficiency loss	- Advanced light delivery systems (e.g., internal illumination, solar concentrators) - Integration of plasmonic materials and photonic crystals to enhance light harvesting and uniformity	[355,372]
Mass Transfer	- Limitation in gas–liquid–solid interactions affecting reaction rates - Poor mixing leading to concentration gradients	- Enhanced mixing strategies to improve contact between phases - Structured catalysts and membrane-integrated systems to facilitate better mass transfer	[373,374]

**Table 9:** Quantitative assessment of key challenges proposed solutions in scaling up photocatalytic hydrogen production.

Scaling-Up Factor	Challenges	Quantitative Metrics	Proposed Solutions	References
Light Delivery Efficiency	- Light Scattering. - Poor penetration in slurries. - Limited light absorption by photocatalyst.	- Single LED to optical fiber efficiency: ~91% evanescent wave utilization. - TiO_2_-coated optical fibers enhance degradation by 32%.	- Optical fibers with reduced TiO_2_ patchiness (0.034 cm^2^/cm^2^). - Optimized interspace distance (114.3 nm) for evanescent waves.	[375,376]
Reactor Design and Photocatalyst Loading	- Low photocatalyst mass-loading. - Reactor inefficiencies. - Catalyst detachment.	- Mass loading of g-C3N4-POFs up to 100–1000× higher than conventional reactors. - Photocatalyst coated optical fibers improved micropollutant degradation by 4×.	Bundled 150 optical fibers for higher quantum efficiency and scalable production.	[377,378]
Surface Area Utilization	- Limited catalyst loading. - Light scattering losses. - Inefficient pollutant degradation. - Catalyst leaching. - High energy consumption.	- High mass loading g-C_3_N_4_ embedded in metamaterial porous polymer fibers (100–1000× higher than traditional coatings). - 4× higher degradation rates compared to slurry reactors. - Maintains photocatalytic activity for 20+ cycles. - No catalyst leaching. - Reduced energy-per-order (EEO) compared to slurry reactors	- Metamaterial porous polymer fibers for efficient light delivery and increased reaction sites. - Photocatalyst immobilization for long-term stability. - Energy-efficient fiber-based reactor designs	[377,379]
Biofouling and Catalyst Stability	- Biofilm accumulation on photocatalytic surfaces reduces efficiency and leads to fouling. - Controlling biofilm growth in enclosed water systems is challenging due to light delivery limitations.	- UV-C SEOFs (side-emitting optical fibers) inhibited biofilm formation at ≥10 µW/cm^2^ (265–275 nm). - UV-A and UV-B SEOFs were ineffective and even increased EPS (Extracellular Polymeric Substance) accumulation, leading to more fouling.	- Low-fluence UV-C SEOFs enable continuous surface disinfection, preventing biofilm accumulation. - Subtractive engineering approach enhances UV-C side-emission, improving biofilm control in confined spaces.	[380,381]
Hydrogen Evolution Reaction (HER) Efficiency	- Electron-hole recombination losses reduce reaction efficiency. - Conventional systems suffer from low quantum yield due to inefficient light absorption.	- Quantum yield increased by nearly 2× when reactor length was doubled. - Photocatalytic H_2_O_2_ production improved by 60× compared to slurries, demonstrating superior light utilization. - HER efficiency increased significantly using g-C_3_N_4_ and ITO-modified polymer optical fibers.	- Evanescent wave enhancement using optical fibers improves light utilization. - Dual optical membrane fiber systems enable stable photocatalysis with enhanced oxygen delivery and light absorption.	[376,378,382]
Water Source and Impurities	- Organic pollutants act as reactive species scavengers, reducing oxidation efficiency. - Background organic matter (e.g., WWTP effluent) significantly decreases photocatalytic degradation rates.	- Organic matter in secondary wastewater effluent (17 mg TOC/L) inhibited photocatalysis. - In the presence of WWTP effluent, BPA removal rates decreased by 52% for electrospun TiO_2_ fibers, compared to 91% for suspended TiO_2_.	- Coupled adsorption-photocatalysis systems enhance contaminant capture and oxidation efficiency. - Porous electrospun fibers increase surface area, improving pollutant access to photocatalytic sites. - TiO_2_ immobilization mitigates organic interference, enhancing stability and reusability.	[379]
Chemical Stability of Photocatalysts	- Potential long-term performance degradation.	- Photocatalytic performance sustained over 20 cycles, with no structural loss of optical fibers.	- High-mass loading photocatalysts. - Roll-to-roll fabrication for scalable and stable production.	[377]
Photoelectrochemical (PEC) Integration	- High energy cost due to inefficient PEC designs.	- Geometric space capacity: 2670 m^2^/m^3^ (>25× higher than flat PEC electrodes). - Photocurrent density: 0.2 mA/cm^2^. - Hydrogen production rate: 15× higher than standard reactors.	- ITO- and g-C_3_N_4_-coated polymer optical fiber optoelectrode improves electron transfer and efficiency.	[378]
Flexible Fiber-Based Reactor Designs	- Need for scalable and adaptable configurations for industrial applications. - Current reactor designs suffer from low energy efficiencies due to light attenuation.	- >6000% larger surface area than flat glass electrodes. - >300% better incident photon-to-current efficiency.	- Flexible Perovskite-Nafion-ITO fiber optoelectrodes for efficient light-driven PEC water purification and hydrogen production.	[383]

**Table 10:** Key innovations in bio-hybrid photocatalytic systems.

Category	Innovation	Significance	Example Applications	References
Material Integration	Combination of photocatalysts with engineered proteins and synthetic biological components	Enhanced synergy between biological selectivity and inorganic stability	Large-scale hydrogen production systems	[409]
Hybrid System Design	Bio-hybrid architectures incorporating light-harvesting complexes with multiple catalytic sites	Increased efficiency and robustness under visible light conditions	Artificial photosynthesis setups for clean energy	[410]
Electron Transfer Pathways	Biomimetic electron transfer mechanisms inspired by natural processes	Improved quantum efficiency and system longevity	Sustainable solar-to-hydrogen energy conversion	[411]
Performance Optimization	Development of systems with high solar-to-hydrogen conversion rates and operational stability	Breakthrough in achieving industrial scale feasibility	Scaled-up photocatalytic energy production plants	[412]
Advanced Architectures	Architectures combining multiple photocatalytic centers for distributed light utilization	Uniform energy conversion and improved system durability	Decentralized renewable energy systems	[413]

## Data Availability

This study did not involve the creation or analysis of new data; therefore, data sharing is not applicable.
